# Student-generated multiple-choice questions enhance deeper learning in dental materials education: a randomized crossover trial

**DOI:** 10.1186/s12909-026-08585-1

**Published:** 2026-01-23

**Authors:** Pankaj Gupta, Karthik Shetty, Heeresh Shetty, Kulvinder Singh Banga

**Affiliations:** 1 Department of Conservative Dentistry and Endodontics, Nair Hospital Dental College, Mumbai, India; 2 Department of Conservative Dentistry and Endodontics, Manipal College of Dental Sciences Mangalore, Manipal Academy of Higher Education, Manipal, Karnataka 576104 India

**Keywords:** Active learning, Cognitive development, Deeper learning, Dental education, Dental materials, Multiple-choice questions, Randomized controlled trial

## Abstract

**Background:**

Dental materials education poses unique challenges due to the complex integration of scientific principles with clinical applications. Traditional teaching methods often fail to promote deep conceptual understanding. This study investigated whether the process of generating multiple-choice questions (MCQs) by students could enhance deeper learning, knowledge application, and critical thinking in dental materials education.

**Methods:**

A prospective, randomized crossover study was conducted among second-year dental students (*n* = 64) at the Nair Hospital Dental College, Mumbai. The study comprised of two phases examining different topics of similar difficulty (dental restorative composites and glass ionomer cements). Following the didactic lectures, all students completed the pre-tests and were randomly allocated to the intervention (MCQ generation activity with faculty-moderated discussion) or control group (additional study time). After a washout period of two weeks, the groups were crossed over. The primary outcome was the difference in post-test performance between the groups. The secondary outcomes included performance across cognitive domains and student feedback. Data were analyzed using paired and independent t-tests, with mixed-effects models for crossover analysis.

**Results:**

Sixty-two students completed both study phases. The intervention group demonstrated significantly higher post-test scores compared to controls in both phases: Phase 1 (approximately 76.8% ± 7.6% vs. 65.3% ± 8.9%, *P* < 0.001, Cohen’s d = 1.38) and Phase 2 (approximately 74.5% ± 8.1% vs. 63.9% ± 9.4%, *P* < 0.001, Cohen’s d = 1.22). The largest improvements were observed in application/analysis questions (Cohen’s d = 1.89 and 1.57 for phases 1 and 2, respectively). Mixed-effects analysis confirmed significant intervention effects (F = 42.36, *P* < 0.001) without period or carryover effects. Student feedback was positive, with approximately 87.1% of the students reporting enhanced understanding.

**Conclusions:**

Student-generated MCQs offer an effective and scalable strategy for fostering deeper learning in dental material education. This process encourages active engagement, supports higher-order cognitive development, and can be seamlessly integrated into existing curricula with minimal resource investment. This approach can potentially enhance both academic outcomes and clinical readiness in dental education.

**Registry:**

Clinical Trial Registry of India (ctri.icmr.org.in/), registration number- CTRI/2025/07/091029 Registration Date-16th July 2025.

**Supplementary Information:**

The online version contains supplementary material available at 10.1186/s12909-026-08585-1.

## Background

Dental materials education constitutes a fundamental component of dental curricula globally; however, it poses distinct pedagogical challenges owing to its interdisciplinary nature, encompassing chemistry, physics, materials science, and clinical application [1]. Traditional pedagogical approaches in dental education often rely on didactic lectures, laboratory demonstrations, and practical exercises. However, these methods do not necessarily foster the deeper conceptual understanding that is essential for effective clinical problem-solving [2].

The notion of “deeper learning” transcends simple factual recall, encompassing critical thinking, conceptual understanding, and the capacity to apply knowledge across diverse contexts [3–5]. In dental materials education, deeper learning requires students to connect microscopic properties with macroscopic behaviours and ultimately with clinical outcomes [6, 7]. This complex integration presents a significant challenge for traditional pedagogical approaches.

Multiple-choice questions (MCQs) are predominantly used as assessment tools in health professions education [8]. Nonetheless, the potential of MCQs as learning instruments warrants further investigation. The testing effect, or retrieval practice, is well-documented in cognitive science literature, demonstrating that active retrieval of information enhances long-term retention compared to passive study methods [9–11]. Building upon this foundation, we hypothesize that student-generated MCQs could enhance knowledge transfer and application in dental materials education, similar to Maggio et al.‘s finding that repeated questioning facilitated knowledge transfer in medical education contexts [12].

The pedagogical benefits of student-generated questions have been investigated across multiple academic fields [13–16]. When students create their own assessment items, they engage in higher-order cognitive processes such as analysis, synthesis, and evaluation. Zaidi et al. demonstrated that medical students who generated MCQs exhibited enhanced critical thinking skills and deeper comprehension of course material [17]. Similarly, a systematic review by Touissi et al. after examining seventeen articles reaches the conclusion that MCQ generation activity seems to be a useful tool for medical students learning [18].

Although these findings from medical education are promising, they cannot be directly extrapolated to dental materials education due to several key differences. First, dental materials science requires unique integration of physicochemical principles with biomechanical properties and clinical handling characteristics—a more complex interrelationship than typically encountered in basic medical sciences [19, 20]. Second, dental materials education involves both theoretical understanding and tactile manipulation skills, creating distinctive learning challenges [21]. Third, the rapid evolution of dental materials technology requires students to develop not only factual knowledge but also conceptual frameworks that allow critical evaluation of novel materials, a competency not extensively studied in previous MCQ-generation research.

Addressing the common criticism that MCQs primarily promote rote memorization rather than conceptual understanding [22], when students generate their own MCQs across different levels of the cognitive domain of Bloom’s taxonomy (knowledge, comprehension, application, and analysis), they must engage with material at multiple cognitive depths [23]. The process of creating plausible distractors (incorrect options) requires students to analyze common misconceptions and subtle distinctions between concepts—a task necessitating deeper understanding than simple memorization [24]. As Gonzalez-Cabezas et al. demonstrated, student-created questions often target higher cognitive levels than faculty-created questions, suggesting that the generation process itself elicits deeper thinking [25].

This study addresses these knowledge gaps by implementing and evaluating a structured intervention involving student-generated MCQs specifically designed to promote a deeper understanding of dental materials concepts. This research builds upon cognitive science principles and incorporates elements of Bloom’s taxonomy as applied to dental education by Gonzalez et al. to ensure that learning activities target various cognitive domains [25].

The primary objective of this investigation was to evaluate whether the process of generating and discussing MCQs enhances dental students’ deeper learning of materials science concepts compared to traditional teaching methods alone. We hypothesized that students participating in MCQ generation activities would demonstrate superior conceptual understanding, knowledge retention, and application abilities compared to those receiving conventional instruction only.

## Materials and methods

### Study design and setting

A prospective, interventional, randomized crossover trial was conducted at the Department of Conservative Dentistry and Endodontics at the Nair Hospital Dental College, Mumbai, India, between July and August 2025. Approval from the Institutional Ethics Committee (EC-213/CONS/ND113/2024) was sought before the commencement of the study. The study was conducted in accordance with the principles of the Declaration of Helsinki. The study was registered with the Clinical Trial Registry of India (CTRI) with the registration number- CTRI/2025/07/091029 dated 16th July 2025.

### Participants

All second-year undergraduate dental students (*n* = 72) were briefed about the purpose of the study and were invited to participate. The inclusion criteria were enrolment in a second-year dental program, attendance at both study sessions, and provision of written informed consent. Exclusion criteria included prior extensive experience with MCQ generation activities, concurrent participation in other educational research, or inability to attend both study phases.

### Sample size calculation

Based on pilot data and expecting a large effect size (Cohen’s d = 0.8), with α = 0.05, and power = 80%, a minimum of 26 participants per group was required. To account for potential dropouts and ensure adequate power, we recruited 32 participants per group. Sample size calculations were performed using G*Power 3.1.9 software.

### Randomization and allocation

Participants were randomized using computer-generated random sequences in blocks of four, stratified by gender. Allocation concealment was maintained using sealed, opaque, and sequentially numbered envelopes. Due to the nature of the intervention, participants and faculty could not be blinded to group allocation.

### Study protocol

The study comprised two phases, each examining different dental material topics of equivalent difficulty.

- Phase 1: Dental restorative composites.
- Phase 2: Glass ionomer cements.

Phase 1 Protocol

1. All participants attended a standardized 60-minute didactic lecture on dental restorative composites delivered by a senior faculty member using identical slides and content.
2. Following the lecture, all participants completed a pre-test comprising 30 validated MCQs covering various cognitive domains according to Bloom’s taxonomy.
3. The students were randomly allocated to two groups:

- Intervention Group (*n* = 32): Participated in MCQ-generation activity.
- Control Group (*n* = 32): Received additional study time with standard learning materials.

4. The intervention group received structured templates and standardized guidance for generating nine MCQs (three each at the knowledge, comprehension, and application levels) based on lecture content. They were provided with reference materials and faculty support during a 120-minute session.
5. A faculty-moderated group discussion was conducted for the intervention group to review, critique, and refine student-generated MCQs. Faculty members actively guided students toward deeper conceptual understanding through Socratic questioning, clarified scientific concepts, corrected misconceptions, and challenged oversimplification.
6. One week after the initial lecture, all participants completed a post-test comprising 30 validated MCQs of comparable difficulty to the pre-test, but with different questions.

### Phase 2 Protocol

After a two-week washout period [26], Phase 2 was conducted following an identical protocol but examining glass ionomer cements. The validated MCQs used in Phase 2 were developed using the same rigorous process as Phase 1, including expert review, content validity assessment, and pilot testing to ensure comparable difficulty and discrimination indices. The washout period duration was determined based on educational research on knowledge retention and previous crossover studies in dental education [26]. Groups were crossed over, with the previous control group serving as the intervention group, and vice versa.

**Fig. 1:**
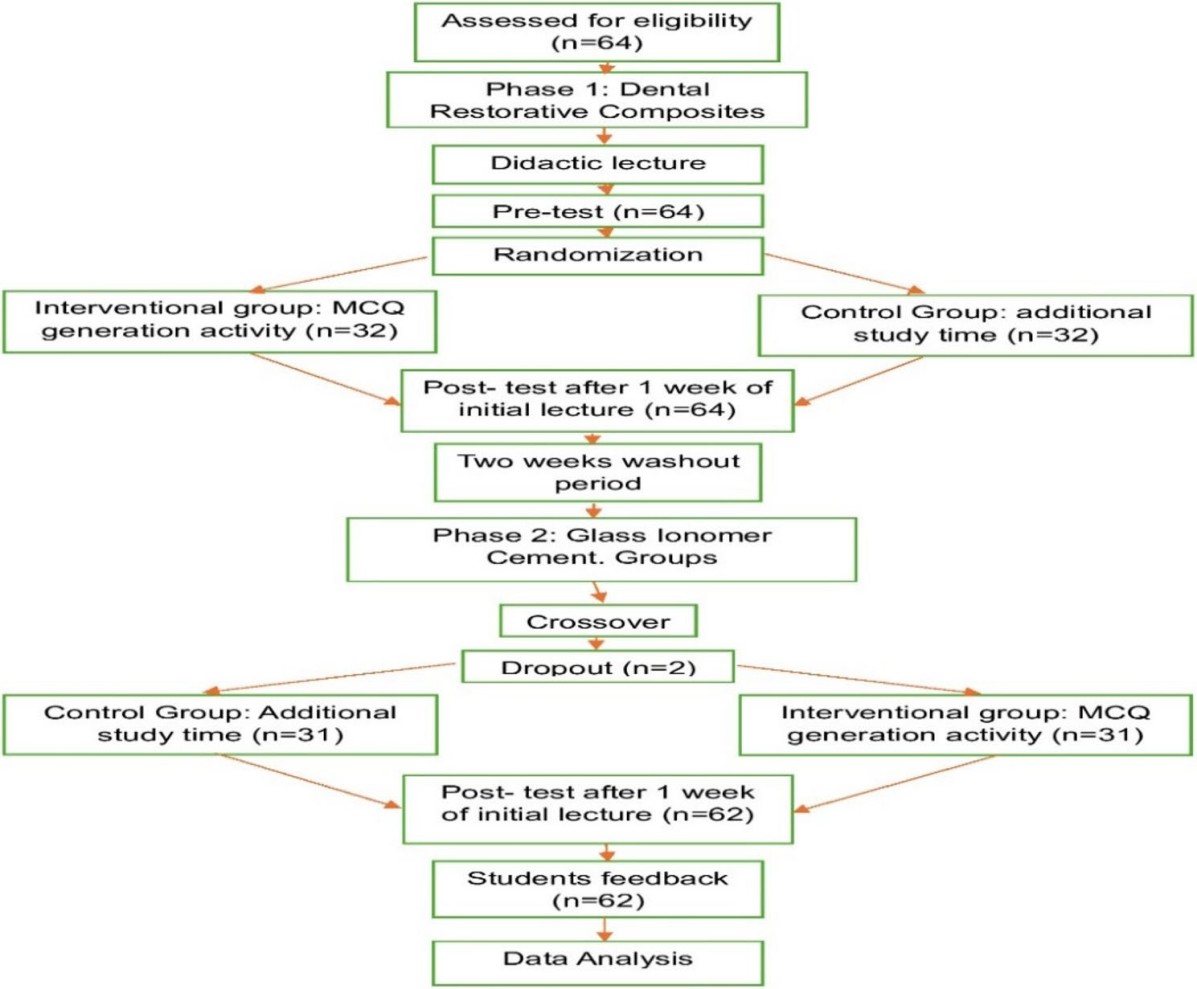
Flowchart of the study design

The study protocol is illustrated in Fig. 1.

### Assessment Tools

#### MCQ development and validation

The pre- and post-test MCQs were developed by a panel of three dental materials experts, each with at least 10 years of teaching experience. Content validity was established through review by two external subject matter experts. Pilot testing on 15 recently graduated students assessed difficulty and discrimination indices, with appropriate modifications made. Acceptable difficulty indices were set between 0.3 and 0.8, and discrimination indices above 0.2 were considered satisfactory.

Both pre- and post-test MCQs in Phase 1 and Phase 2 underwent identical validation procedures. For Phase 2, an independent set of validated MCQs was developed by the same expert panel, maintaining the same cognitive domain distribution (30% knowledge/recall, 35% comprehension, 35% application/analysis) and quality standards as Phase 1.

#### Psychometric comparability between phases

Table 1 A presents the psychometric properties of the MCQs used in both study phases. These data demonstrate that the difficulty indices and discrimination indices were strictly comparable between Phase 1 and Phase 2, confirming the equivalence of assessment instruments across study phases.

**Table 1:** Psychometric properties of MCQs - Phase 1 vs. Phase 2 comparability

Property	Phase 1 (Composites)	Phase 2 (Glass Ionomers)	*P*-value
Difficulty Index - Post-test	0.67 ± 0.09	0.65 ± 0.08	0.421
Difficulty Index - Pre-test	0.52 ± 0.11	0.51 ± 0.10	0.687
Discrimination Index - Pre-test	0.34 ± 0.12	0.33 ± 0.11	0.734
Discrimination Index - Post-test	0.48 ± 0.10	0.46 ± 0.09	0.418
Range of Difficulty Indices	0.30–0.78	0.31–0.79	—
Range of Discrimination Indices	0.21–0.62	0.20–0.61	—

All difficulty and discrimination indices fell within acceptable ranges (difficulty: 0.3-0.8; discrimination: >0.2) for both phases, with no significant differences between phases

#### Assessment of deeper learning

The MCQs in both the pre- and post-tests were categorized based on Bloom’s taxonomy:

- Knowledge/recall (30%).
- Comprehension/understanding (35%).
- Application/analysis (35%).

This distribution emphasized higher order thinking skills assessment.

#### Student feedback collection

After completing both phases, all participants completed a structured feedback questionnaire with closed ended questions with a 5-point Likert scale response and open-ended questions regarding their perceptions of the MCQ generation activity.

#### Statistical analysis

Data analysis was performed using SPSS version 25.0 (IBM Corp., Armonk, NY, USA). Descriptive statistics were calculated for all variables. Normal distribution was assessed using the Shapiro-Wilk test. Paired t-tests were used to compare pre- and post-test scores within groups, and independent t-tests were used to compare scores between groups. Cohen’s d was calculated for effect sizes. The crossover design was analyzed using mixed-effects models to account for period effects, with participants as random effects and treatment, period, and sequence as fixed effects. Intraclass correlation coefficients (ICC) were calculated to assess within-subject correlations. Statistical significance was set at *P* < 0.05. All analyses followed intention-to-treat principles.

## Results

### Participant flow and demographics

Of the 72 students invited, 64 consented to participate. Two students dropped out after Phase 1 due to scheduling conflicts, leaving 62 students (31 in each group after crossover) for the final analysis. Participants included 38 females (61.3%) and 24 males (38.7%), with a mean age of 19.6 ± 1.2 years. No significant differences in baseline characteristics were observed between the initially assigned groups. Table 2 summarizes the demographic details of the study participants.

**Table 2:** Demographic characteristics of participants (*n* = 62)

Characteristic	Value (*n* = 62)
Gender	
Female	38 (61.3%)
Male	24 (38.7%)
Age (years)	
Mean ± SD	19.6 ± 1.2
Range	18–23
Prior Experience with MCQ Generation	
Yes	7 (11.3%)
No	55 (88.7%)

### Phase 1: Dental restorative composites

#### Pre-test scores

Mean pre-test scores demonstrated no significant difference between the intervention group (approximately 52.4% ± 8.7%) and the control group (approximately 53.1% ± 9.2%) (*P* = 0.745), confirming comparable baseline knowledge.

#### Post-test scores

The intervention group showed significantly higher post-test scores (approximately 76.8% ± 7.6%) than the control group (approximately 65.3% ± 8.9%) (*P* < 0.001), with a large effect size (Cohen’s d = 1.38, 95% CI: 0.82–1.94).

#### Performance across cognitive domains

Analysis by question category revealed particularly pronounced differences in the application/analysis questions.

- Knowledge/recall: Intervention approximately 82.4% vs. Control approximately 75.6% (*P* = 0.031, Cohen’s d = 0.83, 95% CI: 0.30–1.36).
- Comprehension: Intervention approximately 78.3% vs. Control approximately 68.9% (*P* = 0.007, Cohen’s d = 1.08, 95% CI: 0.54–1.62).
- Application/analysis: Intervention approximately 69.7% vs. Control approximately 51.4% (*P* < 0.001, Cohen’s d = 1.89, 95% CI: 1.30–2.48).

### Phase 2: Glass ionomer cements

#### Pre-test scores

No significant differences were observed in pre-test scores between the crossover intervention group (approximately 50.9% ± 8.3%) and the control group (approximately 51.7% ± 9.0%; *P* = 0.697).

#### Post-test scores

Consistent with Phase 1, significantly higher post-test scores (approximately 74.5% ± 8.1%) were achieved by the intervention group as compared to the control group (approximately 63.9% ± 9.4%) (*P* < 0.001, Cohen’s d = 1.22, 95% CI: 0.67–1.77).

Table 3 summarizes the pre and post test scores of both the phases for the intervention and control group.

**Table 3:** Comparison of Pre-test and Post-test scores between groups

Group	Pretest Mean ± SD	Post-test Mean ± SD	Mean Difference (95% CI)	*P*-value	Effect Size (Cohen’s d)
Phase 1: Dental Restorative Composites					
Intervention (*n* = 32)	52.4 ± 8.7	76.8 ± 7.6	24.4 (20.7–28.1)	< 0.001	1.38 (0.82–1.94)
Control (*n* = 32)	53.1 ± 9.2	65.3 ± 8.9	12.2 (8.9–15.5)	< 0.001	1.36 (0.80–1.92)
Between-group *P*-value	0.745	< 0.001	-	-	-
Phase 2: Glass Ionomer Cements					
Intervention (*n* = 31)	50.9 ± 8.3	74.5 ± 8.1	23.6 (20.0-27.2)	< 0.001	1.22 (0.67–1.77)
Control (*n* = 31)	51.7 ± 9.0	63.9 ± 9.4	12.2 (8.7–15.7)	< 0.001	1.29 (0.74–1.84)
Between-group *P*-value	0.697	< 0.001	-	-	-

#### Performance across cognitive domains

The pattern of enhanced performance in higher cognitive domains was replicated.

- Knowledge/recall: Intervention approximately 80.1% vs. Control approximately 73.8% (*P* = 0.042, Cohen’s d = 0.74, 95% CI: 0.22–1.26).
- Comprehension: Intervention approximately 76.9% vs. Control approximately 67.2% (*P* = 0.009, Cohen’s d = 1.10, 95% CI: 0.56–1.64).
- Application/analysis: Intervention approximately 66.5% vs. Control approximately 50.7% (*P* < 0.001, Cohen’s d = 1.57, 95% CI: 1.00-2.14).

### Combined analysis

Mixed-effects model analysis confirmed the intervention effect (F = 42.36, *P* < 0.001), while accounting for potential period effects (F = 0.94, *P* = 0.336) and carryover effects (F = 1.22, *P* = 0.274). The intraclass correlation coefficient (ICC) for within-subject correlation was 0.23, indicating an appropriate correlation for crossover design.

Table 4 summarize the performance of the participating students across different cognitive domains for both the phases combined.

**Table 4:** Performance across cognitive domains (Combined Phases)

Cognitive Domain	Intervention Mean ± SD	Control Mean ± SD	Mean Difference (95% CI)	*P*-value	Effect Size (Cohen’s d)
Knowledge/recall	82.4 ± 7.6	75.6 ± 8.9	6.8 (3.1–10.5)	0.031	0.83 (0.30–1.36)
Comprehension	78.3 ± 7.8	68.9 ± 9.3	9.4 (5.6–13.2)	0.007	1.08 (0.54–1.62)
Application/analysis	69.7 ± 8.0	51.4 ± 9.1	18.3 (14.5–22.1)	< 0.001	1.89 (1.30–2.48)

#### Student feedback

##### Quantitative feedback analysis

Responses were based on a 5-point Likert scale (1 = Strongly Disagree, 5 = Strongly Agree):

- Approximately 87.1% agreed or strongly agreed that the activity enhanced their understanding of dental materials concepts (Mean score: 4.31 ± 0.63).
- Approximately 82.3% reported increased engagement with the subject matter (Mean score: 4.17 ± 0.72).
- Approximately 79.0% noted improved ability to identify key concepts and relationships (Mean score: 4.08 ± 0.81).
- Approximately 74.2% indicated enhanced critical thinking skills (Mean score: 3.97 ± 0.88).

Table 5 describes the quantitative analysis of the students feedback about the activity undertaken by them.

**Table 5:** Quantitative analysis of student feedback on MCQ generation activity

Statement	Mean Score* ± SD	Agree or Strongly Agree (%)
The MCQ generation activity enhanced my understanding of dental materials concepts	4.31 ± 0.63	87.1
The activity increased my engagement with the subject matter	4.17 ± 0.72	82.3
Creating MCQs improved my ability to identify key concepts and relationships	4.08 ± 0.81	79.0
The process enhanced my critical thinking skills	3.97 ± 0.88	74.2
Faculty-moderated discussion was valuable for correcting misconceptions	4.42 ± 0.59	90.3
Creating plausible distractors deepened my understanding	4.23 ± 0.67	85.5
I would recommend this activity for other dental materials topics	4.05 ± 0.92	77.4
The activity helped me connect theoretical concepts with clinical applications	3.89 ± 0.94	71.0

*5-point Likert scale: 1 = Strongly Disagree, 5 = Strongly Agree; *n* = 62

#### Thematic analysis of qualitative feedback

Two independent researchers conducted structured thematic analysis of open-ended responses, achieving an inter-rater reliability (Cohen’s kappa) of 0.84:

Table 6 summarizes the thematic analysis of the open-ended feedback questions.

**Table 6:** Themes identified in qualitative analysis of student feedback

Theme	Frequency (%)	Representative Quotation
Enhanced conceptual clarity	53 (85.5%)	“Creating questions about composite polymerization forced me to really understand the chemistry rather than just memorize it.”
Improved identification of knowledge gaps	48 (77.4%)	“I realized I didn’t fully understand the role of coupling agents until I tried to create questions about them.”
Heightened awareness of content importance	45 (72.6%)	“I started recognizing which material properties would actually matter in clinical situations.”
Development of critical thinking	42 (67.7%)	“I started thinking about why incorrect answers about glass ionomer setting reactions were wrong, which helped me understand the acid-base concepts better.”
Increased collaboration and peer learning	39 (62.9%)	“Discussing the questions with faculty and peers revealed misconceptions about filler particles that I didn’t know I had.”

## Discussion

This randomized crossover trial demonstrates that student-generated multiple-choice questions (MCQs) significantly enhance deeper learning in dental materials education. The intervention group consistently outperformed controls across both study phases, with particularly striking improvements in higher order thinking skills. These results suggest that active engagement through assessment content generation consolidates foundational knowledge while developing cognitive flexibility needed for complex clinical applications [27].

The large effect sizes observed—exceeding 1.2 in both phases—carry both statistical and educational significance. In dental education contexts, where conceptual understanding gains are often modest despite intensive instruction, these findings are noteworthy. Effect sizes above 1.2 represent very strong intervention effects [28], indicating that improvements in students participating in MCQ-generation activities were educationally meaningful and likely to translate into noticeable gains in learning outcomes, comprehension, and retention. However, it is important to consider that these large effects might partly reflect the controlled study environment and motivated participant sample, which may not fully represent typical classroom conditions. We anticipate that effect sizes in routine classroom settings would likely be somewhat smaller than those observed here, perhaps in the range of Cohen’s d = 0.8–1.1 rather than the 1.2–1.4 observed in this study. However, even these reduced effect sizes would remain educationally meaningful and justify broader implementation.

Enhanced performance in application- and analysis-level questions represents a particularly significant finding. This pattern suggests that MCQ-generation activities specifically target higher-order thinking skills—a conclusion consistent with Palmer and Devitt’s research showing that student-generated assessment items promoted deeper learning approaches in medical education [29]. The substantial improvement in these cognitive domains (Cohen’s d values of approximately 1.89 and 1.57) indicates that the intervention successfully moved students beyond surface-level memorization toward genuine conceptual understanding. Alternative explanations for these improvements, such as increased study time or heightened motivation from research participation, cannot be entirely ruled out, though the crossover design helps control for such confounding factors.

The results for dental restorative composites carry particular significance given the complexity of this material system. Composites present unique challenges due to their varied composition (resin matrix, fillers, coupling agents), complex polymerization chemistry, and technique-sensitive clinical application [30]. The marked improvement in application/analysis questions suggests that MCQ-generation activities helped students forge meaningful connections between theoretical concepts (degree of conversion, polymerization shrinkage) and clinical implications (marginal adaptation, wear resistance). This bridging of theory and practice represents a crucial competency for restorative dentistry.

Similarly, enhanced understanding of glass ionomer cements in Phase 2 indicates successful navigation of known educational challenges in this area. Glass ionomers involve complex acid-base setting reactions and possess unique properties including fluoride release and chemical adhesion to tooth structures [31]. Student feedback specifically highlighted improved conceptual clarity regarding setting mechanisms and bioactivity—areas typically requiring integration of chemistry, materials science, and clinical knowledge. The consistency of improvements across both material systems suggests that the intervention’s benefits are generalizable across different dental materials topics rather than being material specific.

Student feedback provides valuable insights into how the intervention shaped learning processes. The process of generating plausible distractors appears to have prompted deeper analytical thinking about material properties and behaviors. As Denny et al. observed, creating distractors requires sophisticated discrimination between related concepts [32]. One student’s comment—“Trying to create wrong-but-reasonable answers about composite shrinkage made me think more carefully about the polymerization process than I ever had before”—exemplifies this deeper engagement. The faculty-moderated discussion component likely contributed significantly by enabling immediate misconception correction and understanding reinforcement, consistent with formative assessment best practices [33].

The crossover design strengthens these findings by allowing each student to serve as their own control, reducing variability and ensuring that observed effects stem from the intervention rather than individual differences or topic-related difficulty variations. The absence of significant period or carryover effects indicates consistent intervention benefits independent of exposure sequence. However, the relatively short two-week washout period, while practically necessary, may not have completely eliminated all learning carryover between phases—a limitation that should be considered when interpreting these results.

Compared to other active learning methodologies in dental education, the MCQ-generation approach offers distinct practical advantages. Unlike problem-based learning, which often demands extensive curricular restructuring and significant faculty resources [34], MCQ-generation activities can supplement existing teaching methods with relatively modest time investments. Compared to case-based learning [35], the MCQ approach provides more structured engagement with fundamental scientific principles—particularly valuable for foundational subjects like dental materials. Team-based learning shares similarities with our approach but typically requires more elaborate preparation and in-class facilitation [36]. These comparisons suggest that MCQ-generation may offer an optimal balance between educational effectiveness and implementation feasibility.

From a theoretical perspective, these results support constructivist learning theory, wherein knowledge is actively constructed by learners rather than passively received [37]. MCQ-generation activities represent constructed learning that positions students as producers rather than mere consumers of educational content. The intervention aligns with “assessment as learning” concepts, where assessment activities themselves become powerful learning experiences [38]. The observed improvements in higher order thinking particularly support cognitive science research on retrieval practice and generation effects, where creating MCQs requires students to retrieve, organize, and apply knowledge in ways that strengthen memory networks and facilitate later recall [39]. This supports Karpicke and Blunt’s assertion that retrieval practice produces more learning than elaborative studying with concept mapping [40].

The practical implications for dental education are substantial. MCQ-generation activities represent relatively low-resource interventions readily implementable into existing curricula. They require minimal additional faculty time compared to traditional teaching methods yet produce significant educational benefits. Furthermore, student-generated questions themselves become valuable resources for future cohorts, contributing to growing banks of peer-created assessment items. However, successful implementation requires careful attention to question quality control, adequate faculty training in moderation techniques, and clear guidelines for students to ensure questions target appropriate cognitive levels.

### Sustainability and generalizability considerations

The sustainability of these effects under real-world classroom conditions warrants careful consideration. Several factors may influence the magnitude of intervention effects in routine practice:

#### Controlled study conditions

This study employed standardized protocols, dedicated faculty time for moderation, and research team oversight. In typical educational settings, implementation may lack this level of standardization and resource intensity. Faculty members may have competing demands, larger class sizes might limit individualized discussion time, and the quality control of student-generated questions may vary.

#### Participant motivation

Students participating in research studies often demonstrate heightened motivation and engagement compared to those in standard courses. The presence of pre- and post-tests and awareness of data collection may have enhanced focus and effort. In routine educational practice, students may not maintain this elevated engagement level.asdas

### Implementation recommendations

For practical implementation in dental curricula, we recommend the following approach:

1. Begin with structured templates that prompt students to create questions at different cognitive levels.
2. Provide clear guidelines for constructing effective distractors that reveal common misconceptions.
3. Allocate sufficient time (90–120 min) for faculty-moderated discussion of student-generated questions.
4. Incorporate this activity after traditional instruction but before summative assessments.
5. Create repositories of high-quality student-generated questions for use in future teaching.

This implementation requires minimal resources beyond faculty time for moderation and can be integrated into existing course structures without major curricular reorganization.

## Limitations

Several important limitations warrant consideration and transparent reporting.

### Study scope and generalizability

This single-institution study with a moderate sample size may limit generalizability. Multi-institutional investigations across diverse educational settings, class sizes, and student populations would strengthen external validity and clarify whether findings are context dependent.

### Washout period adequacy

The two-week washout period, while practical within course constraints, may have been insufficient to completely eliminate carryover effects. A longer washout period of approximately 2–3 months or a full semester would have been more ideal to ensure complete dissipation of learning effects between phases. However, such extended periods were not feasible given the compressed timeline of the dental materials curriculum. Students may have retained knowledge, consolidation strategies, or deeper understanding frameworks from Phase 1 that influenced their Phase 2 performance. This represents a potential confounding factor that should be considered when interpreting the crossover results.

### Topic difficulty and content differences

Although both topics (dental restorative composites and glass ionomer cements) were selected for equivalent complexity, we could not completely account for potential inherent difficulty differences between these material systems. Composites involve polymerization chemistry, while glass ionomers involve acid-base reactions. These distinct mechanisms may present different learning challenges or opportunities for some students.

### Assessment of Long-Term retention and clinical transfer

This study assessed only immediate post-test performance conducted one week after instruction. We did not evaluate long-term knowledge retention at 3, 6, or 12 months. More importantly, this study did not assess the transfer of learning to actual clinical practice, laboratory performance, or clinical decision-making.

### Critical knowledge-to-practice gap

The enhanced conceptual understanding observed in this study—measured through improved MCQ performance—may not necessarily translate into improved clinical decision-making, better material selection in patient care, or superior laboratory technique. The ultimate goal of dental materials education is to produce clinically competent practitioners who can make appropriate decisions in real patient care situations. Whether enhanced test performance on application-level questions correlates with actual clinical application remains unknown. This represents a critical gap requiring future investigation, as educational gains in test performance do not automatically guarantee clinical competency or improved patient outcomes.

### Hawthorne effect

The Hawthorne effect—wherein participants change behavior because they know they are being studied—may have contributed to the observed results. Students’ awareness of being observed and data collection procedures may have enhanced engagement and effort beyond typical classroom conditions. However, the crossover design partially mitigated this concern by exposing all participants to both conditions, allowing within-subject comparison.

### Faculty beliefs and expectations

Enthusiasm bias from faculty members who believed in the intervention’s effectiveness might have influenced student engagement and expectations. Faculty members who expected positive results might have unconsciously provided more encouragement or support to the intervention group. Although standardized protocols for faculty interactions were established to minimize this effect, complete elimination of such bias is difficult to achieve in open-label educational research.

### Sample characteristics

The study sample consisted of students willing to participate in educational research, potentially representing a more motivated group than the general dental student population. This sample was relatively homogeneous in age (mean 19.6 years) and educational level (second-year students). Results may not generalize to older learners, students with prior clinical experience, or diverse populations with varying baseline knowledge and learning styles.

## Conclusions

Student-generated MCQs represent an effective pedagogical approach for promoting deeper learning in dental materials education, particularly for complex topics such as dental restorative composites and glass ionomer cements. This randomized crossover trial provides empirical evidence that the process of creating, discussing, and refining MCQs enhances conceptual understanding, critical thinking, and knowledge application in ways that traditional teaching methods alone do not achieve. The intervention was particularly effective in developing higher-order cognitive skills necessary for clinical decision-making in restorative dentistry.

Dental educators should consider incorporating student-generated MCQs as regular components of dental materials curricula to foster deeper engagement with challenging subject areas. The intervention can be implemented with minimal resource requirements while producing educationally meaningful improvements in learning outcomes.

Important Caveat: While this study demonstrates that student-generated MCQs improve test-based measures of conceptual understanding and application, the transfer of these improvements to actual clinical decision-making, patient care quality, and professional competency has not been established. Educators implementing this intervention should view it as supporting deeper learning and conceptual development while recognizing that clinical competency ultimately requires integration of knowledge with practical experience, clinical judgment, and patient-centered decision-making.

### Future directions

Building directly on these findings, we propose a specific follow-up investigation to address unanswered questions about the long-term impact of MCQ-generation activities on dental materials knowledge retention and clinical application. This multi-center, longitudinal study would track three cohorts of dental students over a two-year period:

1. An intervention group engaging in regular MCQ-generation activities across multiple dental materials topics.
2. A comparison group using case-based learning for the same topics.
3. A control group receiving traditional instruction only.

Assessment would occur at multiple time points (immediate, 3 months, 1 year, and 2 years) and would include not only knowledge tests but also practical laboratory assessments and clinical decision-making scenarios. The primary outcome measure would be the transfer of materials science knowledge to preclinical laboratory performance and early clinical decision-making.

### Ethical considerations for future research

Important ethical considerations must be addressed in the proposed multi-center trial. Assigning students to a control group receiving only traditional instruction when evidence suggests MCQ-generation activities may be superior could raise concerns about demoralization bias (where control group students perform worse due to perceived disadvantage) or reversed John Henry effects (where control group students compensate by working harder). To address these ethical concerns while maintaining research integrity, we propose:

1. Offering MCQ-generation workshops to control group students after the primary data collection period, ensuring all participants ultimately benefit from the intervention.
2. Ensuring all groups receive high-quality instruction that meets or exceeds standard educational requirements.
3. Providing detailed informed consent explaining the research rationale, potential benefits and risks, and the importance of control groups in educational research.
4. Implementing regular monitoring to detect any adverse effects on student performance, academic confidence, or psychological wellbeing.
5. Establishing clear stopping rules if the control group demonstrates significantly inferior outcomes that could compromise their educational progress or professional competency development.
6. Conducting interim analyses to assess group differences and ensure ethical conduct throughout the study.

These safeguards would help balance the scientific need for rigorous research with the ethical imperative to ensure all students receive optimal educational experiences.

### Additional research directions

Other promising avenues for future research include:

- Exploring the integration of digital technologies (online platforms, mobile applications) to enhance and scale MCQ-generation interventions.
- Investigating the impact of student-generated questions on clinical decision-making in actual patient care settings.
- Examining faculty perspectives on implementation challenges, required training, and integration with existing curricula.
- Developing quality assessment frameworks and repositories of high-quality student-generated questions that can benefit future cohorts of dental students.
- Investigating whether MCQ-generation skills transfer to other courses and clinical rotations beyond dental materials education.

## Supplementary Information

Supplementary Material 1.

## Data Availability

The datasets generated and analyzed during the course of the current study are available from the corresponding author upon reasonable request.
