# Supplementary material for: Student-generated multiple-choice questions enhance deeper learning in dental materials education: a randomized crossover trial
Source: BMC Med Educ. 2026 Jan 23;26:290. doi: 10.1186/s12909-026-08585-1 (PMC12910890; doi:10.1186/s12909-026-08585-1)
Supplement: Supplementary file 1 — Supplementary Material 1. [file 12909_2026_8585_MOESM1_ESM.docx]

**APPENDIX A: Sample MCQs Across Bloom's Taxonomy Levels**

**Sample 1: Knowledge/Recall Level**

**Question:** Which of the following monomers is the primary resin matrix component in most dental composite resins?

**A)** Polymethyl methacrylate (PMMA)
**B)** Bisphenol A glycidyl methacrylate (BisGMA)
**C)** Polyethylene terephthalate (PET)
**D)** Polyvinyl chloride (PVC)

**Correct Answer:** B

**Cognitive Level Explanation:** This question requires students to recall factual knowledge about composite resin chemistry without requiring analysis or application.

**Sample 2: Comprehension/Understanding Level**

**Question:** When composite resin polymerizes, the conversion of monomer to polymer causes the material to shrink toward the light source. What is the best explanation for why this directional shrinkage occurs?

**A)** The light energy causes the material to expand outward
**B)** Covalent bonds form between monomers, pulling the material toward the greatest light intensity
**C)** The filler particles repel each other during polymerization
**D)** The material naturally flows away from the light source during setting

**Correct Answer:** B

**Cognitive Level Explanation:** This question requires students to explain the mechanism underlying polymerization shrinkage. Students must demonstrate understanding of the relationship between bond formation and material contraction, moving beyond simple recall to explain a concept.

**Sample 3: Application/Analysis Level**

**Question:** A dentist is preparing a deep Class II cavity on tooth #30 with walls that are relatively perpendicular to the occlusal surface. Which of the following composites and filling techniques would best minimize restoration failure due to marginal microleakage?

**A)** Bulk-fill composite with incremental placement in thin layers to maximize bond strength
**B)** Conventional composite with a single large increment to minimize oxygen inhibition
**C)** Hybrid composite with self-etch adhesive to accelerate polymerization
**D)** Micro-filled composite with mechanical retention locks to reduce stress

**Correct Answer:** A

**Cognitive Level Explanation:** This question requires students to analyze clinical variables (cavity geometry, material properties, shrinkage stress, marginal integrity) and apply their understanding of polymerization shrinkage mechanics to select the most appropriate material and technique. Students must evaluate multiple factors and synthesize knowledge to solve a realistic clinical problem.

**Sample 4: Knowledge/Recall Level (Faculty-Created Test Item)**

**Question:** At what pH does glass ionomer cement complete its setting reaction?

**A)** pH 2-3
**B)** pH 4-5
**C)** pH 6-7
**D)** pH 8-9

**Correct Answer:** B

**Cognitive Level Explanation:** This factual question requires recall of the specific pH range at which glass ionomer acid-base reactions reach completion.

**Sample 5: Comprehension/Understanding Level (Student-Generated)**

**Question:** Why does a glass ionomer cement that is exposed to water during its initial setting period typically exhibit improved clinical longevity compared to one that is completely isolated and kept dry?

**A)** Water increases the degree of polymerization
**B)** Moisture is essential for the acid-base reaction to proceed to completion
**C)** Water initiates additional cross-linking of the polymer matrix
**D)** Dry conditions cause the material to crystallize prematurely

**Correct Answer:** B

**Cognitive Level Explanation:** This question requires students to understand the dependency of glass ionomer setting on moisture availability. Rather than simply recalling that glass ionomers are moisture-sensitive, students must explain the mechanistic reason why moisture is actually beneficial for complete setting—a deeper level of comprehension.

**Sample 6: Application/Analysis Level (Student-Generated)**

**Question:** A patient has multiple existing glass ionomer restorations that have been in place for 5 years. At the recall appointment, one restoration shows evidence of surface erosion and ditching at the gingival margin. Before replacing this restoration, the dentist considers various material options. Based on understanding of material properties and clinical performance, which material would be most appropriate for this high-erosion-risk area?

**A)** Another glass ionomer, unchanged, as it has proved adequate in other areas
**B)** A resin-modified glass ionomer or composite-reinforced ionomer to combine glass ionomer advantages with improved wear resistance
**C)** A conventional resin composite to maximize esthetic properties regardless of durability
**D)** An amalgam restoration to ensure maximum longevity despite esthetic concerns

**Correct Answer:** B

**Cognitive Level Explanation:** This question requires students to analyze multiple variables: observed clinical failure mode (erosion/ditching), material properties relevant to erosion resistance, the trade-offs between aesthetic and durability properties, and the clinical indication for different materials. Students must synthesize knowledge of glass ionomer limitations, understand how resin-modified formulations address these limitations, and make a clinically defensible decision—all hallmarks of higher-order cognitive application.
